# The Relationship between Minority Stress and Depressive Symptoms in the LGBTQA Population from Poland

**DOI:** 10.3390/ejihpe13060076

**Published:** 2023-06-08

**Authors:** Aleksandra Cisek, Aleksandra M. Rogowska

**Affiliations:** Institute of Psychology, University of Opole, 45-052 Opole, Poland

**Keywords:** depression symptoms, LGBTQA, minority stress, sexual and gender minority status

## Abstract

The cross-sectional study examines minority stress and depression symptoms regarding various sexual and gender minority (SGM) identities in lesbian, gay, bisexual, transgender, queer, and asexual (LGBTQA) individuals from Poland. The online survey was conducted among 509 people. Participants aged between 18 and 47 (*M* = 22.39, *SD* = 4.78). Gender identity included 262 cisgender women, 74 cisgender men, 31 transgender women, 53 transgender men, and 89 nonbinary individuals. Sexual identity comprises 197 bisexual, 150 homosexual, 78 pansexual, 33 asexual, 21 undefined, 14 heterosexual, 9 demisexuals, 6 queer, and 1 sapiosexual individual. The Daily Heterosexist Experiences Questionnaire (DHEQ) and the Center for Epidemiologic Studies Depression Scale—Revised (CESD-R) were used to measure minority stress and depression symptoms, respectively. Among LGBTQA participants, 99.80% declared minority stress at least once during the past year. In particular, vicarious trauma was experienced in 99.80% of participants, vigilance in 95.87%, harassment and discrimination in 80.35%, stress related to the family of origin in 69.16%, and to gender expression in 68.76% of respondents. Depression symptoms were found in 62.50% of respondents. Significantly higher rates of depression and minority stress were presented in dual than single SGM individuals. Binomial logistic regression showed that such sources of minority stress as vigilance, harassment, and gender expression could predict depression symptoms. Therefore, prevention and intervention programs should be designed for the LGBTQA population focusing on coping with these sources of minority stress, especially among those of dual SGM identity.

## 1. Introduction

Studies indicate that lesbian, gay, bisexual, transgender, queer, and asexual people (LGBTQA) experience discrimination, victimization, and stigmatization because of their gender and sexual identity, a primary source of minority stress [1,2,3]. According to the minority stress model [4,5,6,7], chronic minority stress leads to several somatic and psychiatric health issues in the minority population [8]. Indeed, several studies showed an increased risk of mental disorders among sexual and gender minority (SGM) groups compared to cisgender heterosexual people [9,10,11,12,13,14,15,16,17,18,19,20,21,22]. In particular, high minority stress increases perceived stress, anxiety, depression and suicidality, and post-traumatic stress disorder (PTSD), worsening subjective wellbeing [9,10,23,24,25,26,27]. Especially, multiple minority people (including those belonging simultaneously to ethnic, race, sexual and gender minorities) experience extremely high discrimination, stigma, and fear of rejection [28]. The Biopsychosocial Minority Stress Framework [29] shows how sexual minority status leads to minority stress and adverse health behavioral factors, including immune dysregulation and heightened psychological stress. Individual differences (e.g., identity centrality, concealing, relationship quality) and cross-sectional identities (e.g., race, ethnicity, age) can play a moderating role in this model. In addition, multiple discrimination can increase the risk of depression, as research conducted in European countries has shown [30]. A recent systematic review confirmed that harassment and victimization and low levels of social and family support contribute to a higher risk of depression in sexual minority adults compared to heterosexuals [31].

The prevalence of depression differs depending on gender identity. In the gender minority sample, the prevalence of depressive symptoms was 52% whereas much lower rates were found for the gender-majority population (27% in cisgender women and 25% in cisgender men) [12]. Borgogna et al. [22] showed that college students with transgender and gender nonconforming identities (such as lesbian, gay, bisexual, and queer identities) reported significantly higher depression symptoms than their counterparts with cisgender identities. Transgender and gender-diverse adolescents with nonheterosexual sexual orientations were two to three times more likely to experience adverse mental health outcomes, as compared to their heterosexual peers who are questioning their gender [32]. Moreover, a younger age may be related to even higher depression rates [19,33]. Depression risk may also be higher among dual SGM participants (than single sexual or gender minority) [22,28,30,34], bisexual persons (compared to LG) [13,20,22,35,36], and among females (than males) [14,37,38]. However, the relationship between distress and the gender of minority samples is ambiguous. For example, lesbians had the lowest rates of mood disorders than gays and bisexuals in the national representative sample of sexual minority people in the United States [39]. Consistent and pronounced mood disorder was predicted in sexual minority men rather than women. In addition, the highest risk of depression was found among US bisexuals of both genders. Furthermore, a large difference in depression rates was found between heterosexual and pansexual participants and small between heterosexuals and gays or lesbians [22].

The frequency of depression symptoms and minority stress dimensions will be examined in the present study in a large SGM sample from Poland, one of the most conservative countries in Europe, in which most citizens declare to belong to the Catholic religion. The country’s context and culture may be related to the strength of stereotypes and prejudices against minorities, which contribute to minority stress and mental health. Furthermore, we will explore the diversity in gender and sexual identity concerning depression and minority stress. Consistent with the previous literature, we hypothesize that high minority stress and depression will be presented in dual SGM individuals [22,28,30,34] (bisexual and pansexual people) [13,20,22,35,36], and women [14]. The associations between depression as an explained variable and such predictors as SGM identity and minority stress will be examined using logistic regression analysis. Age will be added to the regression model as a confounder to explain depression symptoms among SGM participants fully. This study’s results should be a base for preparing appropriate prevention and intervention programs for target groups of LGBTQA people in Poland.

## 2. Materials and Methods

### 2.1. Procedure

The cross-sectional study was conducted in Poland between July 2021 and February 2022 using an online Google Forms survey. The invitation to this study was disseminated via private and open groups on Facebook support and educational groups for people of different sexual and gender identities: *LGBT Polska* (LGBT+ Poland), *Grupa Wsparcia dla Osób Transpłciowych* (Support Group for Transgender People), *Sekcja Edukacji Seksualnej* (Sexual Education Section), *Aseksualni Polska* (Asexual People Poland), *Demiseksualni Polska* (Demisexual People Poland), as well as to Instagram users (who openly introduced themselves as members of the LGBTQIA+ community) and through private messages to members of the online groups mentioned. In some online communities on the social network, it was necessary to obtain permission from the administration to share the survey, so requests for permission were sent to administrators. After approval from the groups’ administrations, the survey was made available to their members. Study participants completed an anonymous online survey consisting of psychological questionnaires and close-ended questions. All subjects gave informed, voluntary consent to participate in this study and were told that they could withdraw their consent to participate at any time. The University Research Ethics Committee of Opole University approved this study’s protocol, and this study’s procedures were designed in accordance with the Declaration of Helsinki. Among 520 respondents, 11 did not meet the inclusion criteria (minimum 18 years old and either noncisgender or nonheterosexual identity status). The final sample included 509 people diverse in gender and sexual identities.

### 2.2. Measures

The Daily Heterosexist Experiences Questionnaire (DHEQ) was developed by Balsam et al. [9] to measure the minority stress among Lesbian, Gay, Bisexual, and Transgender (LGBT) people. We used five scales (with seven items in each) of the DHEQ (vigilance, harassment and discrimination, gender expression, vicarious trauma, and family of origin) in a Polish adaptation [40]. A 6-point Likert response scale was used (from 0 = did not happen/not applicable to me to 5 = it happened and bothered me extremely) for each of the 35 items to describe minority stress over the past 12 months. The higher the score (range 0–175), the higher the level of minority stress it indicates. The internal consistency (Cronbach’s α) was 0.81, 0.81, 0.81, 79, and 0.78 for vigilance, harassment and discrimination, gender expression, vicarious trauma, and family of origin subscales, respectively, and 0.90 for the total score of the DHEQ.

The Center for Epidemiologic Studies Depression Scale—Revised (CESD-R) measures such symptoms defined by the American Psychiatric Association’s Diagnostic and Statistical Manual (DSM-V) for a major depressive episode, such as sadness, anhedonia, guilt, fatigue, suicidal ideation, poor appetite, sleep, concentration, and agitation [41,42,43]. We used the CESD-R in the Polish adaptation [44] and a 5-point Likert response scale (0 = not at all/less than 1 day, 4 = nearly every day for 2 weeks). The total score of the CESD-R ranges from 0–80 (the higher score, the more severe depression), and the risk of major depressive disorder (MDD) is recognized with the cut-off point of 27 [45]. The reliability (Cronbach’s α) of the CESD-R was 0.95 in the current sample.

The sociodemographic information was also collected in the survey, including gender identity (with options: cisgender women, cisgender men, transgender women, transgender men, nonbinary, other), sexual identity (asexual, bisexual, heterosexual, homosexual, pansexual, other), age (number of years), education (primary, secondary, vocational, still studying, higher), place of residence (village, city with up to 50,000 inhabitants, city with up to 150,000 inhabitants, city with over 150,000 inhabitants, urban agglomeration), relationship status (married, partner relationships, single), coming out to family (“does anyone in your family know about your sexual or gender identity?”; yes/no), coming out to friends (“does anyone of your friends know about your sexual or gender identity?”; yes/no), coming out to acquaintances (“does anyone of acquaintances know about your sexual or gender identity?”; yes/no).

### 2.3. Participants

The study sample included 509 adults, diverse in sexual and gender identity, with an average age of 22 (ranging from 18 to 47 years, *M* = 22.39, *SD* = 4.78). Among participants prevailed cisgender women, bisexuals, those with partner relationship status, of secondary education, and living in a city with over 150,000 inhabitants (Table 1). In the sample, 33.99% (*n* = 173) were included in the gender minority group (comprising nonbinary and transgender participants), while 66.01% (*n* = 336) declared cisgender identity. Heterosexual identity presented 2.75% (*n* = 14) of individuals, while 97.25% (*n* = 495) were included in sexual minority sample (including asexual, bisexual, demisexual, homosexual, pansexual, queer, sapiosexual, and undefined participants). Most of the respondents declared coming out to friends.

### 2.4. Statistical Analysis

A frequency and percentage were calculated for categorical variables (demographics, depression, and minority stress). The prevalence of depression and minority stress was compared between people with single and dual SGM status, using Pearson’s χ^2^ test of independence and φ as effect size. To check the parametric properties of depression and minority stress as a continuous variables we used mean (*M*), standard deviation (*SD*), skewness, and kurtosis. Since skewness and kurtosis ranged between ± 1.5 and the sample size was quite large (*n* = 509), the parametric statistics were used in the next steps. Differences between single and dual minority groups in depression and minority stress were assessed using the independent samples student’s *t*-test, with Cohen’s *d* for effect size. A one-way ANOVA with Bonferroni posthoc test was conducted to examine differences in depression and minority stress between groups representing various SGM identities. The effect size for ANOVA was assessed by η^²^*_p_*. The binomial logistic regression was performed to find predictors of depression symptoms (using a cut-off of 27) among such variables as age, gender, minority status, and minority stress scales. The bootstrapping technique based on 5000 successful resamplings was applied to increase the accuracy of the results. All statistical analyses were conducted using the JASP ver. 0.16.1.0. software for Windows.

## 3. Results

### 3.1. Prevalence of Depression and Minority Stress in the SGM Population

First, we used the cut off of 27 to examine the proportion of participants with depression symptoms. In the total sample, 318 people (62.50%) reported depression symptoms. Significantly more participants from the dual SGM sample reported depression symptoms (71.1%) than those from the single SGM group (58.3%), Χ^2^(1) = 8.39, *p* = 0.004, φ = 0.13, OR = 1.81.

Almost all study participants (99.80%) were exposed to at least one of five heterosexist experiences during the past year. In particular, exposure to minority stress concerning vicarious trauma was reported in 99.80% of individuals, vigilance in 95.87%, harassment and discrimination in 80.35%, a family of origin in 69.16%, and gender expression in 68.76% of the sample (Table 1). More dual (99.37%) than single (94.28%) SGM people experienced vigilance, Χ^2^(1) = 7.15, *p* = 0.008, φ = 0.12, OR = 2.26. Harassment and discrimination were found significantly more frequently in people with dual (86.79%) than single (77.43%) SGM identification, Χ^2^(1) = 6.07, *p* = 0.014, φ = 0.02, OR = 0.65. Gender expression was more frequent in the dual (94.34%) than single (57.15%) SGM sample, Χ^2^(1) = 70.43, *p* < 0.001, φ = 0.37, OR = 2.53. However, vicarious trauma was found similarly often in both SGM groups, single (99.71%) and dual (100.00%), Χ^2^(1) = 0.46, *p* = 0.500, φ = 0.03, OR = 0.31. Minority stress from family was significantly more frequent among individuals with dual (78.62%) than single (64.86%) SGM identity, Χ^2^(1) = 9.70, *p* = 0.002, φ = 0.14, OR = 0.69.

On average, participants bothered with four various heterosexist experiences during the past year (*M* = 4.14, *SD* = 0.97). All five such experiences during the last 12 months were declared by 45.38% of participants (*n* = 231); four such stressful events were found in 31.04% (*n* = 158), three cases among 17.09% (*n* = 87), two incidents in a sample of 27 people (5.31%), one was reported in five participants (0.98%), and only one person was not exposed to any one source of minority stress (0.20%). The student’s *t*-test showed significantly more minority stressful experiences in dual (*n* = 159, *M* = 4.59, *SD* = 0.67) than single (*n* = 350, *M* = 3.93, *SD* = 1.02) SGM participants, with medium effect size, t(507) = 7.46, *p* < 0.001, Cohen’s *d* = 0.71.

### 3.2. Differences in Depression Symptoms and Minority Stress between Gender Minority Samples

Differences in the total depression score between gender minority groups, including cisgender women (*n* = 262, *M* = 35.95, *SD* = 19.62), cisgender men (*n* = 74, *M* = 28.30, *SD* = 19.41), transgender women (*n* = 31, *M* = 34.36, *SD* = 19.67), transgender men (*n* = 53, M = 38.32, *SD* = 23.06), and nonbinary persons (*n* = 89, *M* = 42.97, *SD* = 20.46) were examined using one-way ANOVA. The results showed significant variance in depression symptoms between gender minority groups, with a small effect size *F*(4, 504) = 5.59, *p* < 0.001, η^²^*_p_* = 0.04. The Bonferroni posthoc test indicated that cisgender women suffer significantly higher depression levels than cisgender men (*p* = 0.040). In addition, nonbinary people showed significantly more depression symptoms than both cisgender men (*p* < 0.001) and women (*p* = 0.047). Transgender women and transgender men did not differ in depression symptoms from any other minority group.

Minority stress levels (total score) differed between gender minority samples, with a moderate effect size *F*(4, 504) = 15.75, *p* < 0.001, η^²^*_p_* = 0.11. In particular, cisgender women (*M* = 1.47, *SD* = 0.61) showed significantly lower minority stress than transgender women (*M* = 1.87, *SD* = 0.80, *p* = 0.046), transgender men (*M* = 1.94, *SD* = 0.85, *p* < 0.001), and nonbinary persons (*M* = 2.09, *SD* = 0.0.98, *p* < 0.001). Furthermore, cisgender men (*M* = 1.45, *SD* = 0.69) presented significantly less minority stress than transgender men (*p* = 0.002) and nonbinary people (*p* < 0.001). However, no significant differences in minority stress were found between cisgender women and men, and also if transgender women were compared with transgender men and nonbinary people were compared with transgender individuals (both men and women).

### 3.3. Differences in Depression Symptoms and Minority Stress between Sexual Minority Samples

Differences in depression scores were also examined between sexual minority groups, including homosexuals (*n* = 150, *M* = 33.08, *SD* = 21.59), bisexuals (*n* = 197, *M* = 35.05, *SD* = 18.89), pansexuals (*n* = 78, *M* = 38.96, *SD* = 19.59), and other sexual identification (*n* = 84, *M* = 41.99, *SD* = 21.68), including asexual, undefined, heterosexual, demisexual, queer, and sapiosexual individuals. The one-way ANOVA indicated that sexual minority groups differ significantly in depression symptoms, *F*(3, 505) = 4.15, *p* = 0.006, η^²^*_p_* = 0.02. The Bonferroni posthoc test showed that only homosexual persons differ significantly from people of other sexual identification (*p* = 0.008). No other significant differences were found between the sexual minority groups.

Considering the total score of minority stress, sexual minority samples differed significantly, as shown in one-way ANOVA, but the effect size was small, *F*(3, 505) = 4.50, *p* = 0.004, η^²^*_p_* = 0.03. The Bonferroni posthoc test indicated that bisexuals (*M* = 1.52, *SD* = 0.68) have significantly lower minority stress than pansexual people (*M* = 1.84, *SD* = 0.85, *p* = 0.013), and those of other sexual identity participants (*M* = 1.80, *SD* = 0.95, *p* = 0.031). Homosexual individuals (*M* = 1.65, *SD* = 0.72) did not differ significantly in minority stress from the other groups of various sexual identities.

### 3.4. Predictors of Depression Symptoms

The Pearson’s correlations were performed initially to examine the association between depression and minority stress among LGBTQA sample. The results showed that depressive symptoms are positively and weakly associated with the overall minority stress scale (the total score of the DHEQ; *r* = 0.32, *p* < 0.001), and dimensions of vigilance (*r* = 0.28, *p* < 0.001), harassment and discrimination (*r* = 0.27, *p* < 0.001), gender expression (*r* = 0.24, *p* < 0.001), vicarious trauma (*r* = 0.14, *p* < 0.01), and family of origin (*r* = 0.17, *p* < 0.001).

The binomial logistic regression was performed for depression symptoms (CESD-R scores > 27). Among the variables included in the model, transgender men were significantly less likely to suffer from depression (cisgender women were a reference group), as shown in Table 2. In contrast, an increased depression risk was found in participants other than homosexual, bisexual, and pansexual identities (*p* < 0.05), but bootstrapping did not support the association. Similarly, although depression risk was decreased in people who came out to family members (*p* < 0.05), the bootstrapping did not confirm this relationship. Strong predictors of depression were three scales of minority stress, namely vigilance, harassment, and gender expression. The regression model explains 20% of depression symptoms among SGM participants, Nagelkerke *R*² = 0.20, χ^2^(492) = 80.17, *p* < 0.001.

## 4. Discussion

### 4.1. Minority Stress in SGM People

The present study showed that minority stress bothered our SGM sample in the past year. Despite advances in rights for LGBTQA people in modern societies worldwide, including Poland, the SGM population still experiences stigma and discrimination [1]. Negative attitudes and an intolerant climate in schools, workplaces, and private social life can lead to discrimination, stigmatization, victimization, and traumatization related to bullying of those with an SGM identity [16,46,47]. LGBTQA people often demonstrate difficulty adapting to a society created mainly by heterosexual people, experiencing everyday heterosexism and minority stress relating to prejudice, expectations of rejection, concealing, internalized homophobia, and hiding [1,2,3].

In the present study, five sources of minority stress were assessed due to scales of DHEQ: vigilance, harassment and discrimination, gender expression, vicarious trauma, and family of origin. Most Polish LGBTQA participants experienced minority stress during the last 12 months. Respondents were bothered with four various heterosexist experiences, on average. In particular, 99.80% of SGM participants reported at least one exposure to minority stress, and only one person in this study sample among 509 participants did not declare anything. The most frequent experience was vicarious trauma (99.80%), vigilance (95.87%), harassment and discrimination (80.35%), a family of origin (69.16%), and gender expression (68.76%). In addition, double SGM individuals reported significantly higher levels of minority stress than the single SGM sample. In particular, compared to single SGM participants, those of dual SGM reported more frequent heterosexism regarding harassment and discrimination, gender expression, and family of origin. However, vicarious trauma and vigilance did not differentiate between groups of various SGM statuses. Furthermore, people with dual SGM identities have reported a significantly higher number of various minority stressors than those with a single identity.

The present study aligns with previous research performed in Spain among lesbian, gay, bisexual, transgender, and queer (LGBTQ+) individuals [2]. Nearly all Spanish participants have been exposed to at least one heterosexist experience (assessed by the DHEQ scale) during the past year. The most frequently reported were vicarious trauma (99.8%), vigilance (93.8%), discrimination or harassment (84.2%), and isolation (81.7%). The highest exposure to heterosexist experiences showed transgender participants and those of sexual minority identification [2]. Similarly, in our study, cisgender women reported significantly lower minority stress (total score) than transgender women, transgender men, and nonbinary persons. In addition, cisgender men were less likely to declare minority stress than transgender men and nonbinary individuals. Comparing the differences in heterosexist experiences between sexual minority samples, bisexuals demonstrated significantly lower levels of minority stress than pansexual people and those of other sexual identity participants.

### 4.2. Depression in LBTQA People from Poland

The present study examined the prevalence of depression and differences between individuals of various sexual and gender identities in a large LGBTQA sample from Poland. Most SGM participants in our study experienced depression symptoms (62.50%). The depression rates found in this study are higher than those among gender minorities representing US young adults (52%) [12] and LGBTQ+ people from Spain (47%) [2]. However, higher rates of mild-to-severe depression were reported in SGM adolescents from the USA (70.1%) [19] than among Polish LGBTQA participants. The inconsistency between present and previous studies in prevalence rates may be related to cultural differences in the degree of acceptance of sexual and gender diversity. Poland is a conservative country, with most people declaring belonging to the Catholic religion. Therefore, intolerance of LGBTQA people is high and stoked by the current government of law and justice, which can explain higher depression rates among LGBTQA adults from Poland compared to those from Spain or the USA [2,12]. However, both the current and previous studies show that a significantly higher depression risk is presented among people representing SGM than among heterosexual and gender-conforming individuals [14,16,17,20,21,34]. Therefore, prevention and intervention programs should be developed for the LGBTQA population.

Svensson and Frost [33] suggested that LGBT people do not form a homogeneous group, although they share similar experiences related to belonging to a minority population. We found differences in depression scores between various groups of gender and sexual minority participants. In particular, nonbinary people showed significantly more depression symptoms than cisgender people, which is consistent with the previous study [19]. Poteat et al. [19] indicated a higher rate of depression in SGM individuals compared to those with heterosexual and cisgender identities. In our study, transgender participants did not differ from any other gender minority sample (cisgender, nonbinary).

In contrast, in the Spanish minority sample, higher rates of depression symptoms were found in transgender and gender-diverse individuals (TGD; 65.2%) than in cisgender participants (41.4%) [2]. Furthermore, among the cisgender sample in this study, women presented higher depression levels than men. The present result is in line with previous research. Women demonstrate higher depression levels than men in the general population [11]. Among adolescents with sexual minority identification, females reported more depressive symptoms than males [14]. Therefore, biological gender may play a significant role in developing depression independent of minority status. The prevention and intervention programs should include the female sex as a risk factor for depression.

Considering sexual minority groups, homosexuals showed significantly lower levels of depression than the sample with other sexual identification (including asexual, undefined, heterosexual, demisexual, queer, and sapiosexual individuals). Previous studies indicate that bisexual people score higher on depression than homosexuals [48,49]. Other studies showed a higher risk of depression and suicidality among bisexual persons than lesbians and gays [13,20,35]. Furthermore, Jacmin-Park et al. [13] found higher depression levels among bisexual and asexual individuals. In a recent study, Ronzón-Tirado et al. [2] showed that depression was less frequent among gays and lesbians (39.5%) compared to people of other sexual identities (bisexual, pansexual, asexual, demisexual, and queer; 57.4%). Borgogna et al. [22] found large differences in depression between heterosexual and pansexual participants and slight differences between heterosexuals and gays or lesbians. In our sample of SGM people, only 14 individuals declared heterosexual identification, so we did not compare the heterosexual group separately. The bisexual sample did not differ significantly from other SGM identities. However, it is difficult to compare any study since various sample sizes were presented for each SGM subsample and distinct SGM groups for comparison. However, all previous and current studies indicate a high diversity in depression among various SGM population groups. Further research in more representative groups for different SGMs is needed to clarify the issue of discrepancies in depression symptoms.

There is also the interaction between gender and sexual identity and decreasing mental health and wellbeing in the LGBTQA population. Higher depression rates were found in this study in dual than single SGM people (71.1% vs. 58.3%, respectively), which is in line with other studies [22,34]. Therefore, the double SGM population should be a target group for mental health prevention and intervention programs.

Furthermore, the logistic regression model showed that gender and sexual identity might predict depression. In particular, lower rates of depression were found in transgender men and people coming out to the family. At the same time, a higher risk is more likely among people other than homosexual, bisexual, and pansexual identities. However, it is essential to note that the significance level for these associations was low (*p* < 0.05), and bootstrapping did not confirm it. Therefore, more research in larger groups representing particular SGM identities should be performed in the future to verify the present associations. Irrefutable evidence indicated that high levels of minority stress in such dimensions as vigilance, harassment, and gender expression were the strongest and positive predictors of depression in this study. However, all variables included in the regression model explained only 20% of depression variance. Therefore, the other variables not considered in this research may play a more crucial role in depression development in the LGBTQA population. Further studies could examine the other variables, such as perceived stress, coping with stress, anxiety, emotional intelligence, negative emotionality, or personality traits (e.g., neuroticism, introversion), to find the most critical variables for depression among SGM individuals.

### 4.3. Implication of this Study

There are several implications of this study. First, the target groups for prevention and intervention programs are people from the LGBTQA population. In particular, individuals with dual identity (sexual and gender) and also people with such sexual identification as asexual, demisexual, queer, sapiosexual, and undefined are at higher risk of depression. Therefore, depressive symptoms should be screened systematically in the LGBTQA population. Those LGBTQA individuals with a high risk of minority stress, depression, and suicidality should be treated, for example, using cognitive-behavioral therapy (CBT). A recent study showed that a Mindfulness-Based Queer Resilience (MBQR) intervention can effectively mitigate the negative effects of minority stress and improve mental and sexual health among sexually minority men, and also can be recommended [50]. Pachankis [[51]] developed a transdiagnostic therapeutic model specific to LGB and proposed several treatment principles for transdiagnostic minority stress interventions.

The present study showed gender expression is related to increased depression risk but coming out to family decreases depression symptoms in SGM participants. A previous study suggests that increasing family social support is an important preventive factor for people belonging to gender minorities [52]. Wong et al. [53] showed that greater instrumental support from family and connection to social network can significantly reduce minority stress and depression in a sample of young homosexual men. Other findings indicate that gay and bisexual men relied primarily on other LGB individuals, whereas people of other sexual identity (heterosexual women and men, lesbians, and bisexual women) relied primarily on their families in seeking major support [54]. Moreover, results from a large international population study (in 13 countries) showed that psychiatric morbidity is associated with sexual minority status, and this relationship was mediated in part by perceived openness to family, but only among women [15].

Diamond and Aley [55] suggest that a key target for intervention is change in insufficient social safety as a primary cause of stigma-related health disparities among a minority population. Social support, in particular, from family and friends may be also crucial to coping with minority stress and preventing depression symptoms, as suggests numerous research [56,57,58]. Since the indirect relationship between sexual minority stigma and depression leads through resilience and family support in the SGM population, both building up resilience of and cultivating family support should be prioritized in the implementation of social policies [59]. Systemic therapy, with participation of minority adults and youth and their parents and close friends, should be helpful as a basic therapy.

Campus climate is also one of the significant factors related to mental health of minority populations [1,60], therefore school administrators should improve the school climate in their educational institutions at each level (primary, secondary, high schools, colleges, and universities). In addition, school education should provide evidence-based knowledge about gender and sexual identities, foster tolerance and positive attitudes toward gender and sexual diversity from an early age, and build healthy communities. In addition, support groups online or stationary could be organized in school, work, and other places to allow LGBTQA people to meet and talk about their problems, building a supportive social network in the SGM community.

Since higher rates of loneliness are reported among SGM than heterosexuals, as suggested by previous studies [61], interventions are needed at the individual and societal level to decrease the loneliness experienced by sexual minorities. Based on a review of theories and evidence on minority stigma and stress, cognitive behavioral therapy and antibullying policies are recommended as part of public health interventions to effectively target stigma and improve the mental and physical health of LGBT young people [62]. Studies show that cognitive behavioral intervention should focus on tackling stigma and alleviate loneliness at an individual level [63,64].

Coping with harassment should be a main aim for prevention and intervention to improve mental health of SGM participants, as suggests the present study. Zheng [65] showed that emotion-oriented and cognitive coping processes can be helpful in decreasing minority stress and improving mental health, including depression. Techniques focusing on increasing self-esteem and acceptance of sexual identity should be performed to decrease internalized homophobia, increase coping with minority stress, and reduce mental health problems [66]. Among coping strategies, behavioral avoidance was associated with worsening depressive symptoms, while seeking social support in adults was associated with lower levels of depression among adolescents from 11 European countries who experience peer victimization at school [67]. Research among gender minority participants confirms that both minority stressors and passive coping were associated with a higher likelihood of suicidality [68]. Therefore, avoidant coping and especially refraining from doing nothing is not effective in reducing the risk of depression, and seeking social support should be trained among potential victims. Furthermore, interventions aimed at improving self-efficacy in emotion-focused coping self-efficacy can be particularly helpful in reducing the negative impact of stigma on physical health among minority people [69]. Additionally, self-compassion can act as a protective factor against minority stress and should be trained in the LGBTQA population [23].

The sense of coherence (as a dimension of resilience) has a protective effect and significantly reduces the risk of depression among people with SGM and should be included in counseling programs [70]. A review study demonstrated evidence that the social and community context (e.g., neighborhood and built environment, health and health care, education, and economic stability) contributes to mental health and wellbeing through discrimination and other minority stress in SGM people [71]. A Social Affective Neuroscience Model of Risk and Resilience in Adolescent Depression [72] suggests that resilience building in sexual and gender minority youth should target interventions in families, peer groups, and schools to promote acceptance and support, and reduce internalized stigma by enhancing skills, managing responses to social threats, and adopting an SGM identity. As mental distress is influenced by social factors specific to SGM, including barriers to social integration, interventions among minority populations may be aimed at addressing their social needs [73]. Specifically, this should include facilitating mental health counseling, increasing LGBTQ friends, mentoring LGBTQ newcomers, and joining an LGBTQ community.

Politicians, local government officials, NGOs, and social workers should also pay special attention to health disparities and more frequent homelessness among sexual and gender minorities [74,75]. Individuals with high levels of internalized transphobia and low levels of gender identity appearance congruence are more likely to meet diagnostic criteria for depression, as suggested a study among transgender and gender-nonconforming adolescents and young adults [76]. More frequent experiences of victimization and lack of social support among bisexual women, gay men, and younger lesbian women partially explain the mental health treatment disparities based on sexual orientation, as suggested by a prospective study from Sweden [74]. Therefore, health-related intervention programs should be tailored differently for specific sexual and gender identities [77].

Research showed that some cultural variables (e.g., ethnic identity, familism) and gender can be helpful in understanding how to manage minority stressors [78]. For example, familism buffered the positive association between acculturative stress and depressive symptoms among a sample of students of Mexican ancestry in the USA. Furthermore, ethnic identity search and commitment moderated the association between perceived discrimination and depressive symptoms and was also a protective factor for women but not for men [78]. Since minority stress is related to cultural variables in ethnic minorities, specific cultural variables could also be determined for gender and sexual minority populations, but more research is necessary to support this speculation. This Polish study among an SGM population and its clinicians [79] showed that those with a bisexual sexual identity were afraid of powerlessness in the face of the social situation of LGBTQA people. On the other hand, among homosexuals, the greatest concern was caused by psychotherapists’ attempts to change their sexual orientation. However, both groups considered support in the process of accepting their sexual orientation and coping with problems resulting from functioning in a prejudiced society as the most desirable goals of psychotherapy.

To summarize, although some differences were found between diverse gender and sexual minority samples, almost all SGM participants experience high levels of minority stress. Therefore, coping with minority stress should be offered to all SGM populations. In addition, strategies based on relaxation and reducing anxiety should also be commonly used in LGBTQA people.

### 4.4. Strengths and limitations of the Study

This study’s main strength is the relatively large sample size of LGBTQA people from Poland. However, we used a conventional online survey, so the current sample may not represent the whole LGBTQA population in the country and other countries worldwide. Future crosscultural studies should include more representative samples from various countries to compare depression and minority stress across countries and cultures. Furthermore, since the self-report screening questionnaire may be related to some measurement bias, the prevalence of depression symptoms may be overvalued. In addition, we used a cross-sectional study, preventing causal relationships between variables. A longitudinal study could verify the relationships between depression and minority stressors among the LGBTQA population.

## 5. Conclusions

This study found alarmingly high rates of minority stress and depression symptoms among LGBTQA participants from Poland. Although some differences were presented between individuals with diverse sexual and gender identities, the whole SGM population needs professional help focused on mental health issues. The prevention strategies should be implemented in schools and mass media, while intervention programs require a multidisciplinary approach to decrease depression among LGBTQA people. In particular, strategies of coping with such sources of minority stress as vigilance, harassment, and gender expression should be offered to LGBTQA people, especially those with dual SGM identities.

## Figures and Tables

**Table 1:** Participant characteristics.

Variable	Categories	*n*	%
Gender identity	Cisgender Men	74	14.54
	Cisgender Women	262	51.47
	Nonbinary	89	17.49
	Transgender Men	53	10.41
	Transgender Women	31	6.09
Sexual identity	Asexual	33	6.48
	Bisexual	197	38.70
	Demisexual	9	1.77
	Heterosexual	14	2.75
	Homosexual	150	29.47
	Pansexual	78	15.32
	Queer	6	1.18
	Sapiosexual	1	0.20
	Undefined	21	4.13
Dual identiy	Sexual and gender minority	159	31.24
Education	Primary	39	7.66
	Secondary	192	37.72
	Vocational	11	2.16
	Still studying	169	33.20
	Higher	98	19.25
Place of residence	Village	77	15.13
	City with up to 50,000 inhabitants	85	16.70
	City with up to 150,000 inhabitants	61	11.98
	City with over 150,000 inhabitants	180	35.36
	Urban agglomeration	106	20.83
Relationship status	Married	11	2.16
	Partner relationships	261	51.28
	Single	237	46.56
Coming out	To family	379	74.46
	To friends	493	96.86
	To acquaintances	452	88.80
Depression	Symptoms	318	62.48
Minority stress	Vigilance	488	95.87
	Harassment	409	80.35
	Gender expression	350	68.76
	Vicarious trauma	508	99.80
	Family of origin	352	69.16

**Table 2:** Binomial regression model for depression symptoms in sexual and gender minority adults.

						Wald Test		95% bca CI	
Variable	*b*	*SE b*	β	AOR	*z*	χ^2^(1)	*p*	LL	UL
**Intercept**	2.01	0.96	2.06	7.48	2.10	4.40	0.036	−0.49	4.72
**Age**	−0.04	0.02	−0.19	0.96	−1.69	2.85	0.091	−0.09	0.02
**Gender identity (cisgender women ref.)**									
Cisgender men	−0.55	0.32	0.34	0.58	−1.70	2.89	0.089	−1.22	0.13
Transgender women	−0.44	0.48	0.37	0.65	−0.91	0.83	0.361	−1.41	0.74
Transgender men	−0.93	0.40	0.39	0.39	−2.33	5.41	0.020	−1.72	−0.07
Nonbinary	−0.14	0.37	−0.08	0.87	−0.38	0.15	0.703	−0.87	0.62
**Sexual identity (homosexual person as a reference)**									
Bisexual	0.42	0.26	−0.01	1.52	1.61	2.61	0.106	−0.14	0.91
Pansexual	0.59	0.35	−0.55	1.81	1.67	2.80	0.094	−0.16	1.33
Other sexual identity	0.77	0.38	−0.44	2.15	2.03	4.13	0.042	−0.05	1.49
**Coming out (“No” as a reference)**									
Family	−0.50	0.25	−0.93	0.61	−1.97	3.86	0.049	−1.02	0.04
Friends	−0.55	0.74	−0.14	0.58	−0.74	0.54	0.461	−14.38	1.18
Acquaintances	−0.76	0.39	0.42	0.47	−1.94	3.75	0.053	−1.64	0.04
**Minority stress (continuous variable)**									
Vigilance	0.27	0.10	0.59	1.31	2.71	7.32	0.007	0.04	0.47
Harrasment	0.34	0.13	0.77	1.40	2.52	6.34	0.012	0.06	0.62
Gender expression	0.36	0.14	−0.50	1.43	2.57	6.60	0.010	0.05	0.63
Vicarious trauma	−0.07	0.11	−0.55	0.93	−0.69	0.47	0.493	−0.31	0.16
Family of origin	−0.01	0.12	−0.76	0.99	−0.08	0.01	0.933	−0.25	0.23

Note. CO = coming out, bca = bias corrected accelerated bootstrapping, CI = confidence interval, LL = lover level, UL = upper level.

## Data Availability

Dataset is available in open access at OSF: Rogowska, A.M. (1 May 2023). Minority stress among Polish adults. https://doi.org/10.17605/OSF.IO/Z8HJE.
